# A simple introduction to Markov Chain Monte–Carlo sampling

**DOI:** 10.3758/s13423-016-1015-8

**Published:** 2016-03-11

**Authors:** Don van Ravenzwaaij, Pete Cassey, Scott D. Brown

**Affiliations:** 1 Department of Psychology, University of Groningen, Grote Kruisstraat 2/1, Heymans Building, room H169, Groningen, 9712TS The Netherlands; 2 Department of Psychology, University of Newcastle, University Drive, Aviation Building, Callaghan, NSW 2308 Australia

**Keywords:** Markov Chain Monte–Carlo, MCMC, Bayesian inference, Tutorial

## Abstract

Markov Chain Monte–Carlo (MCMC) is an increasingly popular method for obtaining information about distributions, especially for estimating posterior distributions in Bayesian inference. This article provides a very basic introduction to MCMC sampling. It describes what MCMC is, and what it can be used for, with simple illustrative examples. Highlighted are some of the benefits and limitations of MCMC sampling, as well as different approaches to circumventing the limitations most likely to trouble cognitive scientists.

Over the course of the twenty–first century, the use of Markov chain Monte–Carlo sampling, or *MCMC*, has grown dramatically. But, what exactly is MCMC? And why is its popularity growing so rapidly? There are many other tutorial articles that address these questions, and provide excellent introductions to MCMC. The aim of this article is not to replicate these, but to provide a more basic introduction that should be accessible for even very beginning researchers. Readers interested in more detail, or a more advanced coverage of the topic, are referred to recent books on the topic, with a focus on cognitive science, by Lee (2013) and Kruschke (2014), or a more technical exposition by Gilks et al. (1996).

MCMC is a computer–driven sampling method (Gamerman and Lopes 2006; Gilks et al. 1996). It allows one to characterize a distribution without knowing all of the distribution’s mathematical properties by randomly sampling values out of the distribution. A particular strength of MCMC is that it can be used to draw samples from distributions even when all that is known about the distribution is how to calculate the density for different samples. The name MCMC combines two properties: *Monte–Carlo* and *Markov chain*.^1^ Monte–Carlo is the practice of estimating the properties of a distribution by examining random samples from the distribution. For example, instead of finding the mean of a normal distribution by directly calculating it from the distribution’s equations, a Monte–Carlo approach would be to draw a large number of random samples from a normal distribution, and calculate the sample mean of those. The benefit of the Monte–Carlo approach is clear: calculating the mean of a large sample of numbers can be much easier than calculating the mean directly from the normal distribution’s equations. This benefit is most pronounced when random samples are easy to draw, and when the distribution’s equations are hard to work with in other ways. The Markov chain property of MCMC is the idea that the random samples are generated by a special sequential process. Each random sample is used as a stepping stone to generate the next random sample (hence the *chain*). A special property of the chain is that, while each new sample depends on the one before it, new samples do *not* depend on any samples before the previous one (this is the “Markov” property).

MCMC is particularly useful in Bayesian inference because of the focus on posterior distributions which are often difficult to work with via analytic examination. In these cases, MCMC allows the user to approximate aspects of posterior distributions that cannot be directly calculated (e.g., random samples from the posterior, posterior means, etc.). Bayesian inference uses the information provided by observed data about a (set of) parameter(s), formally the *likelihood*, to update a *prior* state of beliefs about a (set of) parameter(s) to become a *posterior* state of beliefs about a (set of) parameter(s). Formally, Bayes’ rule is defined as

1 $$ p(\mu|D) \propto p(D|\mu) \cdot p(\mu) \label {BayesRule}  $$

where *μ* indicates a (set of) parameter(s) of interest and *D* indicates the data, *p*(*μ*|*D*) indicates the posterior or the probability of *μ* given the data, *p*(*D*|*μ*) indicates the likelihood or the probability of the data given *μ*, and *p*(*μ*) indicates the prior or the a–priori probability of *μ*. The symbol ∝ means “is proportional to”.

More information on this process can be found in Lee (2013), in Kruschke (2014), or elsewhere in this special issue. The important point for this exposition is that the way the data are used to update the prior belief is by examining the likelihood of the data given a certain (set of) value(s) of the parameter(s) of interest. Ideally, one would like to assess this likelihood for every single combination of parameter values. When an analytical expression for this likelihood is available, it can be combined with the prior to derive the posterior analytically. Often times in practice, one does not have access to such an analytical expression. In Bayesian inference, this problem is most often solved via MCMC: drawing a sequence of samples from the posterior, and examining their mean, range, and so on.

Bayesian inference has benefited greatly from the power of MCMC. Even in just in the domain of psychology, MCMC has been applied in a vast range of research paradimgs, including Bayesian model comparison (Scheibehenne et al. 2013), memory retention (Shiffrin et al. 2008), signal detection theory (Lee 2008), extrasensory perception (Wagenmakers et al. 2012), multinomial processing trees (Matzke et al. 2015), risk taking (van Ravenzwaaij et al. 2011), heuristic decision making (van Ravenzwaaij et al. 2014) and primate decision making (Cassey et al. 2014).

While MCMC may sound complex when described abstractly, its practical implementation can be very simple. The next section provides a simple example to demonstrate the straightforward nature of MCMC.

## Example: In–class test

Suppose a lecturer is interested in learning the mean of test scores in a student population. Even though the mean test score is unknown, the lecturer knows that the scores are normally distributed with a standard deviation of 15. So far, the lecturer has observed a test score of a single student: 100. One can use MCMC to draw samples from the *target distribution*, in this case the posterior, which represents the probability of each possible value of the population mean given this single observation. This is an over–simplified example as there is an analytical expression for the posterior ( *N*(100,15)), but its purpose is to illustrate MCMC.

To draw samples from the distribution of test scores, MCMC starts with an initial guess: just one value that might be plausibly drawn from the distribution. Suppose this initial guess is 110. MCMC is then used to produce a chain of new samples from this initial guess. Each new sample is produced by two simple steps: first, a *proposal* for the new sample is created by adding a small random perturbation to the most recent sample; second, this new proposal is either accepted as the new sample, or rejected (in which case the old sample retained). There are many ways of adding random noise to create proposals, and also different approaches to the process of accepting and rejecting. The following illustrates MCMC with a very simple approach called the *Metropolis algorithm* (Smith and Roberts 1993):

1. Begin with a plausible *starting value*; 110 in this example.
2. Generate a new proposal by taking the last sample (110) and adding some random noise. This random noise is generated from a *proposal distribution*, which should be symmetric and centered on zero. This example will use a proposal distribution that is normal with zero mean and standard deviation of 5. This means the new proposal is 110 (the last sample) plus a random sample from *N*(0,5). Suppose this results in a proposal of 108.
3. Compare the height of the posterior at the value of the new proposal against the height of the posterior at the most recent sample. Since the target distribution is normal with mean 100 (the value of the single observation) and standard deviation 15, this means comparing *N*(100|108,15) against *N*(100|110,15). Here, *N*(*μ*|*x*,*σ*) indicates the normal distribution for the posterior: the probability of value *μ* given the data *x* and standard deviation *σ*. These two probabilities tell us how plausible the proposal and the most recent sample are given the target distribution.
4. If the new proposal has a higher posterior value than the most recent sample, then accept the new proposal.
5. If the new proposal has a lower posterior value than the most recent sample, then randomly choose to accept or reject the new proposal, with a probability equal to the height of both posterior values. For example, if the posterior at the new proposal value is one-fifth as high as the posterior of the most recent sample, then accept the new proposal with 20% probability.
6. If the new proposal is accepted, it becomes the next sample in the MCMC chain, otherwise the next sample in the MCMC chain is just a copy of the most recent sample.
7. This completes one *iteration* of MCMC. The next iteration is completed by returning to step 2.
8. Stop when there are enough samples (e.g., 500). Deciding when one has enough samples is a separate issue, which will be discussed later in this section.

**Fig. 1:**
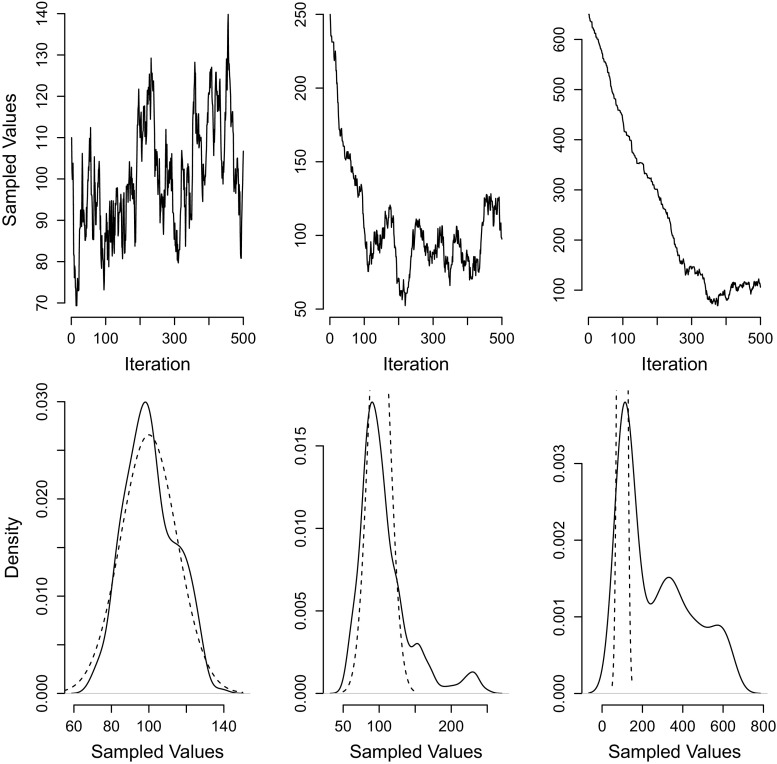
A simple example of MCMC. *Left column:* A sampling chain starting from a good starting value, the mode of the true distribution. *Middle column:* A sampling chain starting from a starting value in the tails of the true distribution. *Right column:* A sampling chain starting from a value far from the true distribution. *Top row:* Markov chain. *Bottom row:* sample density. The analytical (true) distribution is indicated by the dashed line

This very simple MCMC sampling problem only takes a few lines of coding in the statistical freeware program R, available online at cran.r-project.org. Code to do this may be found in Appendix A. The results of running this sampler once are shown in the left column of Fig. 1. These samples can be used for Monte–Carlo purposes. For instance, the mean of the student population test scores can be estimated by calculating the sample mean of the 500 samples.

The top–left panel of Fig. 1 shows the evolution of the 500 iterations; this is the Markov chain. The sampled values are centered near the sample mean of 100, but also contain values that are less common. The bottom–left panel shows the density of the sampled values. Again, the values center around the sample mean with a standard deviation that comes very close to the true population standard deviation of 15 (in fact, the sample standard deviation for this Markov chain is 16.96). Thus, the MCMC method has captured the essence of the true population distribution with only a relatively small number of random samples.

### Limitations

The MCMC algorithm provides a powerful tool to draw samples from a distribution, when all one knows about the distribution is how to calculate its likelihood. For instance, one can calculate how much more likely a test score of 100 is to have occurred given a mean population score of 100 than given a mean population score of 150. The method will “work” (i.e., the sampling distribution will truly be the target distribution) as long as certain conditions are met. Firstly, the likelihood values calculated in steps 4 and 5 to accept or reject the new proposal must accurately reflect the density of the proposal in the target distribution. When MCMC is applied to Bayesian inference, this means that the values calculated must be posterior likelihoods, or at least be proportional to the posterior likelihood (i.e., the ratio of the likelihoods calculated relative to one another must be correct). Secondly, the proposal distribution should be symmetric (or, if an asymmetric distribution is used, a modified accept/reject step is required, known as the “Metropolis–Hastings” algorithm). Thirdly, since the initial guess might be very wrong, the first part of the Markov chain should be ignored; these early samples cannot be guaranteed to be drawn from the target distribution. The process of ignoring the initial part of the Markov chain is discussed in more detail later in this section.

The example MCMC algorithm above drew proposals from a normal distribution with zero mean and standard deviation 5. In theory, any symmetric distribution would have worked just as well, but in practice the choice of proposal distribution can greatly influence the performance of the sampler. This can be visualised by replacing the standard deviation for the proposal distribution in the above example with a very large value, such as 50. Then many of the proposals would be well outside the target distribution (e.g., negative test score proposals!) leading to a high *rejection rate*. On the other hand, with a very small standard deviation, such as 1, the sampler could take many iterations to converge from the starting value to the target distribution. One also runs the risk of getting stuck in *local maxima*: areas where the likelihood is higher for a certain value than for its close neighbors, but lower than for neighbors that are further away.

The width of the proposal distribution is sometimes called a *tuning parameter* of this MCMC algorithm. The fact that the practical performance of the sampler can depend on the value of the tuning parameter is a limitation of the standard Metropolis–Hastings sampling algorithm, although there are many augmented methods that remedy the problem. For example, “auto-tuning” algorithms that adapt the width of the proposal distribution to the nature of the data and distribution (see Roberts & Rosenthal, 2009 for an overview).

The third condition, the fact that initial samples should be ignored as they might be very wrong, deals with a problem known as *convergence* and *burn-in*. For example, suppose the initial guess was one that was very unlikely to come from the target distribution, such as a test score of 250, or even 650. Markov chains starting from these values are shown in the middle and right columns of Fig. 1. Examining the top–middle panel of Fig. 1 shows that the Markov chain initially goes quickly down towards the true posterior. After only 80 iterations, the chain is then centered on the true population mean. Examining the top–right panel of Fig. 1, which has an even more extreme starting point, demonstrates that the number of iterations needed to get to the true population mean — about 300 — is much larger than for better starting points. These two examples make it clear that the first few iterations in any Markov chain cannot safely be assumed to be drawn from the target distribution. For instance, including the first 80 iterations in the top–middle panel or those first 300 iterations in the top–right panel leads to an incorrect reflection of the population distribution, which is shown in the bottom–middle and –right panels of Fig. 1.

One way to alleviate this problem is to use better starting points. Starting values that are closer to the mode of the posterior distribution will ensure faster burn–in and fewer problems with convergence. It can be difficult in practice to find starting points near the posterior mode, but maximum–likelihood estimation (or other approximations to that) can be useful in identifying good candidates. Another approach is to use multiple chains; to run the sampling many times with different starting values (e.g. with starting values sampled from the prior distribution). Differences between the distributions of samples from different chains can indicate problems with burn–in and convergence. Another element of the solution is to remove the early samples: those samples from the non–stationary parts of the chain. When examining again the chains in the top row of Fig. 1, it can be seen that the chain in the top–left has come to some sort of an equilibrium (the chain is said to have “converged”). The chains in the top–middle and –right panel also converge, but only after about 80 and 300 iterations, respectively. The important issue here is that all the samples prior to convergence are *not* samples from the target distribution and must be discarded.

Deciding on the point at which a chain converges can be difficult, and is sometimes a source of confusion for new users of MCMC. The important aspect of burn–in to grasp is the post–hoc nature of the decision, that is, decisions about burn–in must be made after sampling, and after observing the chains. It is a good idea to be conservative: discarding extra samples is safe, as the remaining samples are most likely to be from the converged parts of the chain. The only constraint on this conservatism is to have enough samples after burn–in to ensure an adequate approximation of the distribution. Those users desiring a more automated or objective method for assessing burn–in might investigate the R̂ statistic (Gelman and Rubin 1992).

## MCMC applied to a cognitive model

We are often interested in estimating the parameters of cognitive models from behavioral data. As stated in the introduction, MCMC methods provide an excellent approach for parameter estimation in a Bayesian framework: see Lee (2013) for more detail. Examples of such cognitive models include response time models (Brown and Heathcote 2008; Ratcliff 1978; Vandekerckhove et al. 2011), memory models (Hemmer and Steyvers 2009; Shiffrin and Steyvers 1997; Vickers and Lee 1997) and models based on signal detection theory (SDT: Green & Swets, 1966). Models based on SDT have had a seminal history in cognitive science, perhaps in part due to their intuitive psychological appeal and computational simplicity. The computational simplicity of SDT makes it a good candidate for estimating parameters via MCMC.

Suppose a memory researcher obtains data in the form of hits and false alarms from a simple visual detection experiment. Applying the SDT framework would allow the researcher to understand the data from a process, rather than descriptive (e.g. ANOVA) perspective. That is, estimating the parameters of the SDT model allows the researcher to gain an insight into how people make decisions under uncertainty. SDT assumes that when making a decision under uncertainty one needs to decide whether a certain pattern is more likely to be “signal” (e.g. a sign post on a foggy night) or merely “noise” (e.g. just fog). The parameters of SDT provide a theoretical understanding of how people distinguish between just noise and meaningful patterns within noise: sensitivity, or *d* ^*′*^, gives a measure of the ability of the individual to distinguish between the noise and the pattern; criterion, or *C*, gives a measure of an individual’s bias, at what level of noise are they willing to call noise a meaningful pattern.

One way to estimate SDT parameters from data would be to use Bayesian inference and examine the posterior distribution over those parameters. Since the SDT model has two parameters ( *d* ^*′*^ and *C*), the posterior distribution is bivariate; that is, the posterior distribution is defined over all different combinations of *d* ^*′*^ and *C* values. MCMC allows one to draw samples from this bivariate posterior distribution, as long as one can calculate the density for any given sample. This density is given by Eq. 1: the likelihood of the hits and false alarms, given the SDT parameters, multiplied by the prior of those SDT parameters. With this calculation in hand, the process of MCMC sampling from the posterior distribution over *d* ^*′*^ and *C* is relatively simple, requiring only minor changes from the algorithm in the in–class test example above. The first change to note is that the sampling chain is multivariate; each sample in the Markov chain contains two values: one for *d* ^*′*^ and one for *C*.

The other important change is that the target distribution is a posterior distribution over the parameters. This allows the researcher to answer inferential questions, such as whether *d* ^*′*^ is reliably greater than zero, or whether *C* is reliably different from an unbiased value. To make the target distribution a posterior distribution over the parameters, the likelihood ratio in Step 3 above must be calculated using Eq. 1. A simple working example of such an MCMC sampler for an SDT model may be found in Appendix B.

An important aspect of the SDT example that has not come up before is that the model parameters are correlated. In other words, the relative likelihood of parameter values of *d* ^*′*^ will differ for different parameter values of *C*. While correlated model parameters are, in theory, no problem for MCMC, in practice they can cause great difficulty. Correlations between parameters can lead to extremely slow convergence of sampling chains, and sometimes to non-convergence (at least, in a practical amount of sampling time). There are more sophisticated sampling approaches that allow MCMC to deal efficiently with such correlations. A simple approach is *blocking*. Blocking allows the separation of sampling between certain sets of parameters. For example, imagine the detection experiment above included a difficulty manipulation where the quality of the visual stimulus is high in some conditions and low in others. There will almost surely be strong correlations between the two SDT parameters within different conditions: within each condition, high values of *d* ^*′*^ will tend to be sampled along with high values of *C* and vice versa for low values. Problems from these correlations can be reduced by blocking: that is, separating the propose-accept-reject step for the parameters from the two difficulty conditions (see e.g., Roberts & Sahu, 1997).

## Sampling beyond basic metropolis–hastings

The Metropolis–Hastings algorithm is very simple, and powerful enough for many problems. However, when parameters are very strongly correlated, it can be beneficial to use a more complex approach to MCMC.

### Gibbs sampling

Given a multivariate distribution, like the SDT example above, Gibbs sampling (Smith and Roberts 1993) breaks down the problem by drawing samples for each parameter directly from that parameter’s *conditional distribution*, or the probability distribution of a parameter *given* a specific value of another parameter. An example of this type of MCMC is called Gibbs sampling, which is illustrated in the next paragraph using the SDT example from the previous section. More typically Gibbs sampling is combined with the Metropolis approach, and this combination is often referred to as “Metropolis within Gibbs”. The key is that for a multivariate density, each parameter is treated separately: the propose/accept/reject steps are taken parameter by parameter. This algorithm shows how Metropolis within Gibbs might be employed for the SDT example:

1. Choose starting values for both *d* ^*′*^ and *C*, suppose these values are 1 and 0.5, respectively.
2. Generate a new proposal for *d* ^*′*^, analogous to the second step in Metropolis–Hastings sampling described above. Suppose the proposal is 1.2.
3. Accept the new proposal if it is more plausible to have come out of the population distribution than the present value of *d* ^*′*^, *given the present C value*. So, given the *C* value of 0.5, accept the proposal of *d* ^*′*^ = 1.2 if that is a more likely value of *d* ^*′*^ than 1 for that specific *C* value. Accept the new value with a probability equal to the ratio of the likelihood of the new *d* ^*′*^, 1.2, and the present *d* ^*′*^, 1, given a *C* of 0.5. Suppose the new proposal ( *d* ^*′*^ of 1.2) is accepted.
4. Generate a new proposal for *C*. For this a second proposal distribution is needed. This example will use a second proposal distribution that is normal with zero mean and standard deviation of 0.1. Suppose the new proposal for *C* is 0.6.
5. Accept the new proposal if it is more plausible to have come out of the population distribution than the *C* value, *given the present* *d* ^*′*^ *value*. So, given the *d* ^*′*^ value of 1.2, accept the proposal of *C* = 0.6 if that is a more likely value of *C* than 0.5 for that specific value of *d* ^*′*^. Accept the new value with a probability equal to the ratio of the likelihood of the new *C*, 0.6, and the present *C*, 0.5, given a *d* ^*′*^ of 1.2. Suppose in this case that the proposal for *C* (0.6) is rejected. Then the sample for *C* stays at 0.5.
6. This completes one iteration of Metropolis within Gibbs sampling. Return to step 2 to begin the next iteration.

**Fig. 2:**
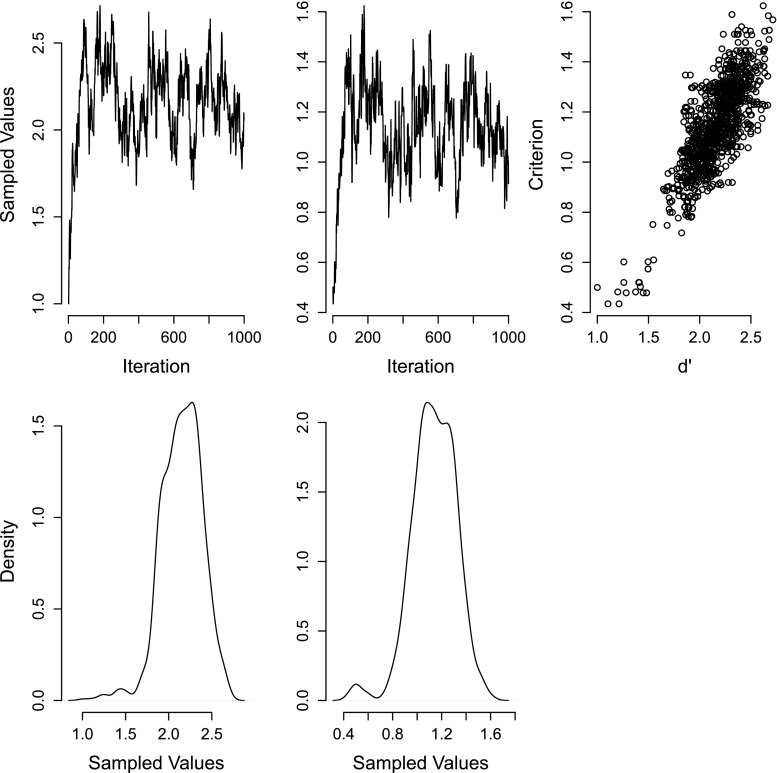
An example of Metropolis within Gibbs sampling. *Left column:* Markov chain and sample density of *d* ^*′*^. *Middle column:* Markov chain and sample density of *C*. *Right column:* The joint samples, which are clearly correlated

R–code for this example can be found in Appendix C. The results of running this sampler are shown in Fig. 2. The left and middle columns show the *d* ^*′*^ and *C* variables respectively. Importantly, the right column shows samples out of the joint posterior, which is a bivariate distribution. It can be seen from this that the parameters are correlated. Such a correlation is typical with the parameters of cognitive models. This can cause a problem for Metropolis–Hastings sampling, because the correlated target distribution is very poorly matched by the proposal distribution, which does not include any correlation between parameters; sampling proposals from an uncorrelated joint distribution ignores the fact that the probability distribution of each parameter differs depending on the values of the other parameters. Metropolis within Gibbs sampling can alleviate this problem because it removes the need to consider multivariate proposals, and instead applies the accept/reject step to each parameter separately.

### Differential evolution

**Fig. 3:**
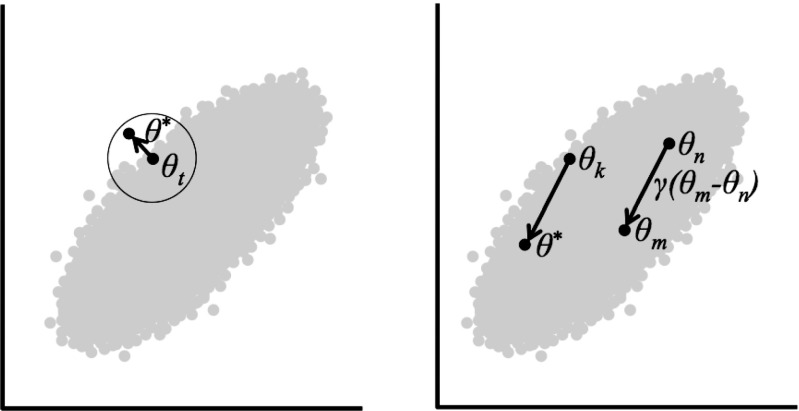
*Left panel:* MCMC sampling using a conventional symmetrical proposal distribution. *Right panel:* MCMC sampling using the crossover method in Differential Evolution. See text for details

The previous section showed how Gibbs sampling is better able to capture correlated distributions of parameters by sampling from conditional distributions. This process, while accurate in the long run, can be slow. The reason is illustrated in the left panel of Fig. 3.

Figure 3 shows a bivariate density very similar to the posterior distribution from the SDT example above. Suppose, during sampling, that the current MCMC sample is the value indicated by *𝜃*_*t*_ in Fig. 3. The MCMC approaches discussed so far all use an uncorrelated proposal distribution, as represented by the circle around *𝜃*_*t*_. This circle illustrates the fact that high and low values of the parameter on the x-axis are equally likely for any different value of the parameter on the y-axis. A problem arises because this uncorrelated proposal distribution does not match the correlated target distribution. In the target distribution, high values of the x-axis parameter tend to co-occur with high values of the y-axis parameter, and vice versa. High values of the y-axis parameter almost never occur with low values of the x-axis parameter.

The mismatch between the target and proposal distributions means that almost half of all potential proposal values fall outside of the posterior distribution and are therefore sure to be rejected. This is illustrated by the white area in the circle, in which proposals have high values on the y-axis but low values on the x-axis. In higher dimensional problems (with more parameters) this problem becomes much worse, with proposals almost certain to be rejected in all cases. This means that sampling can take a long time, and sometimes too long to wait for.

One approach to the problem is to improve proposals and have them respect the parameter correlation. There are many ways to do this, but a simple approach is called “differential evolution” or DE. This approach is one of many MCMC algorithms that use multiple chains: instead of starting with a single guess and generating a single chain of samples from that guess, DE starts with a set of many initial guesses, and generates one chain of samples from each initial guess. These multiple chains allow the proposals in one chain to be informed by the correlations between samples from the other chains, addressing the problem shown in Fig. 3. A key element of the DE algorithm is that the chains are not independent – they interact with each other during sampling, and this helps address the problems caused by parameter correlations.

To illustrate the process of DE–MCMC, suppose there are multiple chains: *𝜃*_1_,*𝜃*_2_,.... The DE–MCMC algorithm works just like the simple Metropolis–Hastings algorithm from above, except that proposals are generated by information borrowed from the other chains (see the right panel of Fig. 3):

1. To generate a proposal for the new value of chain *𝜃*_*k*_, first choose two other chains at random. Suppose these are chains *n* and *m*. Find the distance between the current samples for those two chains, i.e.: *𝜃*_*m*_−*𝜃*_*n*_.
2. Multiply the distance between chains *m* and *n* by a value *γ*. Create the new proposal by adding this multiplied distance to the current sample. So, the proposal so far is: *𝜃*_*k*_+*γ*(*𝜃*_*m*_−*𝜃*_*n*_). The value *γ* is a tuning parameter of the DE algorithm.
3. Add a very small amount of random noise to the resulting proposal, to avoid problems with identical samples (“degeneracy”). This leads to the new proposal value, *𝜃* ^∗^.

Because DE uses the difference between other chains to generate new proposal values, it naturally takes into account parameter correlations in the joint distribution. To get an intuition of why this is so, consider the right panel of Fig. 3. Due to the correlation in the distribution, samples from different chains will tend to be oriented along this axis. For example, very few pairs of samples will have one pair with a higher x-value but lower y-value than the other sample (i.e. the white area in the circle of the left panel of Fig. 3). Generating proposal values by taking this into account therefore leads to fewer proposal values that are sampled from areas outside of the true underlying distribution, and therefore leads to lower rejection rates and greater efficiency. More information on MCMC using DE can be found in ter Braak (2006).

Like all MCMC methods, the DE algorithm has “tuning parameters” that need to be adjusted to make the algorithm sample efficiently. While the Metropolis-Hastings algorithm described earlier has separate tuning parameters for all model parameters (e.g. a proposal distribution width for the *d* ^*′*^ parameter, and another width for the *C* parameter), the DE algorithm has the advantage of needing just two tuning parameters in total: the *γ* parameter, and the size of the “very small amount of random noise”. These parameters have easily–chosen default values (see, e.g., Turner et al., 2013). The default values work well for a very wide variety of problems, which makes the DE–MCMC approach almost “auto–tuning” (ter Braak 2006). Typically, the random noise is sampled from a uniform distribution that is centered on zero and which is very narrow, in comparison to the size of the parameters. For example, for the SDT example, where the *d* ^*′*^ and *C* parameters are in the region of 0.5–1, the random noise might be sampled from a uniform distribution with minimum -0.001 and maximum +0.001. The *γ* parameter should be selected differently depending on the number of parameters in the model to be estimated, but a good guess is $2.38/\sqrt {(2K)}$, where *K* is the number of parameters in the model.

An example of cognitive models that deal with correlated parameters in practice is the class of response time modeling of decision making (e.g. Brown & Heathcote, 2008; Ratcliff, 1978; Usher & McClelland, 2001). As such, they are the kind of models that benefit from estimation of parameters via DE–MCMC. This particular type of MCMC is not trivial and as such a fully worked example of DE–MCMC for estimating response time model parameters is beyond the scope of this tutorial. The interested reader may find an application of DE–MCMC to estimating parameters for the Linear Ballistic Accumulator model of response times in Turner et al. (2013).

## Summary

This tutorial provided an introduction to beginning researchers interested in MCMC sampling methods and their application, with specific references to Bayesian inference in cognitive science. Three MCMC sampling procedures were outlined: Metropolis(–Hastings), Gibbs, and Differential Evolution.^2^ Each method differs in its complexity and the types of situations in which it is most appropriate. In addition, some tips to get the most out of your MCMC sampling routine (regardless of which kind ends up being used) were mentioned, such as using multiple chains, assessing burn–in, and using tuning parameters. Different scenarios were described in which MCMC sampling is an excellent tool for sampling from interesting distributions. The examples focussed on Bayesian inference, because MCMC is a powerful way to conduct inference on cognitive models, and to learn about the posterior distributions over their parameters. The goal of this paper was to demystify MCMC sampling and provide simple examples that encourage new users to adopt MCMC methods in their own research.

## Appendix A: Metropolis R–Code

Code for a Metropolis sampler, based on the in–class test example in the main text. In R, all text after the # symbol is a comment for the user and will be ignored when executing the code. The first two lines create a vector to hold the samples, and sets the first sample to 110. The loop repeats the process of generating a proposal value, and determining whether to accept the proposal value, or keep the present value.

## Appendix B: SDT R–Code

Code for a Metropolis sampler for estimating the parameters of an SDT model. Given a specified number of trials with a target either present or absent, and given (fake) behavioral data of hits and false alarms, the code below evaluates the joint likelihood of SDT parameters, *d* ^*′*^ and *C*. New proposals for both parameters are sampled and evaluated simultaneously.

## Appendix C: Metropolis within Gibbs sampler R–Code

Code for a Metropolis within Gibbs sampler for estimating the parameters of an SDT model. The following code calculates the likelihood of the current *d* ^*′*^ and *C* parameter values (the “posterior.density” function was omitted, but is identical to the one defined in Appendix B. The key difference between the Metropolis sampler in the previous section and the Metropolis within Gibbs sampler in this section is that the proposal and evaluation occurs separately for each parameter, instead of simultaneously for both parameters. The loop over the number of parameters, “for ( j in rownames(samples) )”, allows for parameter *d* ^*′*^ to have a new value proposed and its likelihood evaluated while parameter *C* is held at its last accepted value and vice versa.

**Glossary**

**Accepting**: A proposal value that is evaluated as more likely than the previously accepted value, or that is less likely but is accepted due to random chance. This value then becomes the value used in the next iteration.
**Blocking**: Sampling only a subset of parameters at a time, while keeping the remaining parameters at their last accepted value.
**Burn–In**: Early samples which are discarded, because the chain has not converged. Decisions about burn–in occur after the sampling routine is complete. Deciding on an appropriate burn–in is essential before performing any inference.
**Chain**: One sequence of sampled values.
**Conditional Distribution**: The probability distribution of a certain parameter given a specific value of another parameter. Conditional distributions are relevant when parameters are correlated, because the value of one parameter influences the probability distribution of the other.
**Convergence**: The property of a chain of samples in which the distribution does not depend on the position within the chain. Informally, this can be seen in later parts of a sampling chain, when the samples are meandering around a stationary point (i.e., they are no longer coherently drifting in an upward or downward direction, but have moved to an equilibrium). Only after convergence is the sampler guaranteed to be sampling from the target distribution.
**Differential Evolution**: A method for generating proposals in MCMC sampling. See section “Differential Evolution” for a more elaborate description.
**Gibbs Sampling**: A parameter-by-parameter approach to MCMC sampling. See section “Gibbs Sampling” for a more elaborate description and an example.
**Iteration**: One cycle or step of MCMC sampling, regardless of routine.
**Local maxima**: Parameter values that have higher likelihood than their close neighbors, but lower likelihood than neighbors that are further away. This can cause the sampler to get “stuck”, and result in a poorly estimated target distribution.
**Markov chain**: Name for a sequential process in which the current state depends in a certain way only on its direct predecessor.
**MCMC**: Combining the properties of Markov chains and Monte–Carlo. See their respective entries.
**Metropolis algorithm**: A kind of MCMC sampling. See section “In–Class Test” for a more elaborate description and an example.
**Monte–Carlo**: The principle of estimating properties of a distribution by examining random samples from the distribution.
**Posterior**: Used in Bayesian inference to quantify a researcher’s updated state of belief about some hypotheses (such as parameter values) after observing data.
**Prior**: Used in Bayesian inference to quantify a researcher’s state of belief about some hypotheses (such as parameter values) before having observed any data. Typically represented as a probability distribution over different states of belief.
**Proposal**: A proposed value of the parameter you are sampling. Can be accepted (used in the next iteration) or rejected (the old sample will be retained).
**Proposal Distribution**: A distribution for randomly generating new candidate samples, to be accepted or rejected.
**Rejecting**: A proposal might be discarded if it is evaluated as less likely than the present sample. The present sample will be used on subsequent iterations until a more likely value is sampled.
**Rejection Rate**: The proportion of times proposals are discarded over the course of the sampling process.
**Starting Value**: The initial “guess” for the value of the parameter(s) of interest. This is the starting point for the MCMC sampling routine.
**Target Distribution**: The distribution one samples from in an attempt to estimate its properties. Very often this is a posterior distribution in Bayesian inference.
**Tuning Parameter**: Parameters which influence the behavior of the MCMC sampler, but are not parameters of the model. For example, the standard deviation of a proposal distribution. Use caution when choosing this parameter as it can substantially impact the performance of the sampler by changing the rejection rate.

## Footnotes

1 For these and other definitions, please see the glossary at the end of the paper.

2 For a visualization of Metropolis–Hastings and Gibbs sampling, see http://twiecki.github.io/blog/2014/01/02/visualizing-mcmc/.
